# Supplementary material for: Long-Term Outcomes in Severe Traumatic Brain Injury and Associated Factors: A Prospective Cohort Study
Source: J Clin Med. 2022 Oct 31;11(21):6466. doi: 10.3390/jcm11216466 (PMC9655294; doi:10.3390/jcm11216466)
Supplement: Supplementary file 1 [file jcm-11-06466-s001.zip › jcm-1941004-supplementary.pdf]

Journal of
Clinical Medicine

MDPI

Supplementary Material

The following scales are available as supplementary data: New Injury Severity Score (NISS); Revised Trauma Score (RTS); Trauma and Injury Severity Score (TRISS); New Trauma and Injury Severity Score (NTRISS), Disability Rating Scale (DRS)

The RTS was calculated by adding the values of systolic blood pressure, respiratory rate, and GCS observed at hospital admission to identify the probability of survival of the participants. The RTS score can range from 0 to 7.8408, with higher scores associated with a higher probability of survival. TRISS and NTRISS estimated the physiological severity and the global severity of the trauma. For the calculation of TRISS and NTRISS, the RTS value of the participant's admission to the emergency, the ISS or NISS, the victim's age, and blunt trauma as the type of trauma were considered.

In the DRS, the score varies from 0 (zero) indicates no level of disability; one (1) mild disability; 2 (two) or 3 (three) partial; 4 (four) to 6 (six) moderate; 7 (seven) to 11 (eleven) moderately severe disability; 12 (twelve) to 16 (sixteen) severe; 17 (seventeen) to 21 (twenty-one) extremely severe; 22 (twenty-two) to 24 (twenty-four) vegetative state; 25 (twenty-five) to 29 (twenty-nine) extreme vegetative state and a score of 30 (thirty) for death.[30]

RTS trauma index ranged from 2.628 to 5.967, with a mean of 4.635 (sd = 0.972) and a median of 4.094; TRISS index ranged from 6.6 to 96.1, with a mean of 56.57 (sd = 24.2) and a median of 58.8; NTRISS index ranged from 1 to 96.1, with a mean of 29.7 (sd = 26.43) and a median of 21.2.

The DRS scale showed an important functional improvement, represented by the decrease in the mean score over time, from 18.47 (sd=10.98) at discharge to 4.21 (sd=6.9) at 12 months (Table 1). At hospital discharge, six participants were classified without disability and four with a mild disability; At three months, 23 participants were without disability and 16 with a mild or moderate disability; At 12 months, 42 were without disability and 18 with mild or partial disability. In the two-by-two multiple comparisons, a statistically significant difference was observed between the means of the DRS scale values for all periods ($p < 0.05$).

Table S1. DRS scores after trauma in severe TBI victims with focal injuries.

Scale		Mean (SD)	Median	Min-Max	p-value
DRS	Discharge	18,47 (10,98)	21	0 - 30	<0,001
	3-months	7,97 (8,33)	5	0 - 15	
	6-months	5,49 (7,83)	2	0 - 6	
	12-months	4,21 (6,9)	1	0 - 4	

SD - Standard deviation; Max – maximum value; Min – minimum value; DRS - Disability Rating Scale. Participants during follow-up: 82 participants at discharge, 77 participants at three months, 75 participants at six months and 76 participants at twelve months.

Table S2. Additional factors associated with 14-day mortality in severe TBI victims with focal lesions.

Associated Factors		14-day mortality		p-value
		Yes	No	
RTS	Mean (SD)	4,23 (0,8)	4,89 (0,99)	<0,001
	Min-Max	2,63 - 5,97	2,63 - 5,97	
NISS	Mean (SD)	58,16 (12,7)	50,12 (15,98)	0,003

	Min-Max	27 - 75	14 - 75	
**TRISS**	Mean (SD)	45,86 (22,87)	63,82 (22,18)	<0,001
	Min-Max	11,1 - 96,1	6,6 - 90,1	
**NTRISS**	Mean (SD)	18,52 (18,27)	37,06 (28,31)	<0,001
	Min-Max	1 - 70,3	1,2 - 96,1	

Bpm – beats per minute; GCS – Glasgow Coma Scale; ICU – Intensive Care Unit; ISS – Injury Severity Score; LOS – Length of stay; Max - Maximum value; mg/dL – milligrams per deciliter; Min - Minimum value; NISS – New Injury Severity Score; NTRIS – New Trauma and Injury Severity Score; RR – respiratory rate; RTS – Revised Trauma Score; SD - Standard deviation; TRISS – Trauma and Injury Severity Score.

Table S3. Additional factors associated with functional dependence at 6 and 12 months in severe TBI victims with focal lesions.

**Associated Factors**		**6-months disability**		**p-value**	**12-months disability**		**p-value**
		**Yes**	**No**		**Yes**	**No**	
	Min-Max	3 – 8	3 – 8		3 – 8	3 – 8	
**RTS**	Mean (SD)	4,76 (0,85)	4,86 (1,03)	0,619	4,75 (0,87)	4,85 (1,01)	0,653
	Min-Max	3,57 - 5,97	2,63 - 5,97		3,57 - 5,97	2,63 - 5,97	
	Min-Max	17 - 45	14 - 50		21 - 45	14 - 50	
**NISS**	Mean (SD)	57,26 (15,66)	49,18 (14,59)	0,026	60,93 (13,37)	48,44 (14,83)	0,002
	Min-Max	24 - 75	14 - 75		34 - 75	14 - 75	
**TRISS**	Mean (SD)	55,24 (24,74)	64,84 (21,56)	0,165	50,44 (25,49)	65,53 (20,89)	0,019
	Min-Max	13 - 86,6	11,1 - 96,1		13 - 86,6	11,1 - 96,1	
**NTRISS**	Mean (SD)	27,08 (26,07)	37,69 (27,42)	0,109	21,75 (23,81)	38,75 (27,28)	0,016
	Min-Max	1,2 - 82,3	3,2 - 82,3		1,2 - 82,3	3,2 - 82,3	

Bpm – beats per minute; GCS – Glasgow Coma Scale; ICU – Intensive Care Unit; ISS – Injury Severity Score; LOS – Length of stay; Max - Maximum value; mg/dL – milligrams per deciliter; Min - Minimum value; NISS – New Injury Severity Score; NTRIS – New Trauma and Injury Severity Score; RR – respiratory rate; RTS – Revised Trauma Score; SD - Standard deviation; TRISS – Trauma and Injury Severity Score.

Table S4. Multivariate model for predicting death after severe TBI.

**Variable**	**Estimate**	**p value for model**	**p -value for change over time**
Intercept	-0.064	-	-
Age	0.113	0.500	0.439
ICU LOS	-0.045	**0.016**	0.154
More than one brain lesion	-0.592	0.149	0.065
ISS	0.045	0.064	0.127
GCS	-0.334	**0.008**	0.307

Multivariate model, using automated stepwise selection for the most significant variables. The following variables were included: Age, more than one radiographic lesion on CT imaging, length of stay (LOS) in ICU, ISS at admission and GCS at admission. Correlation between each variable and change over time was analyzed using repeated measures with generalized estimating equations.

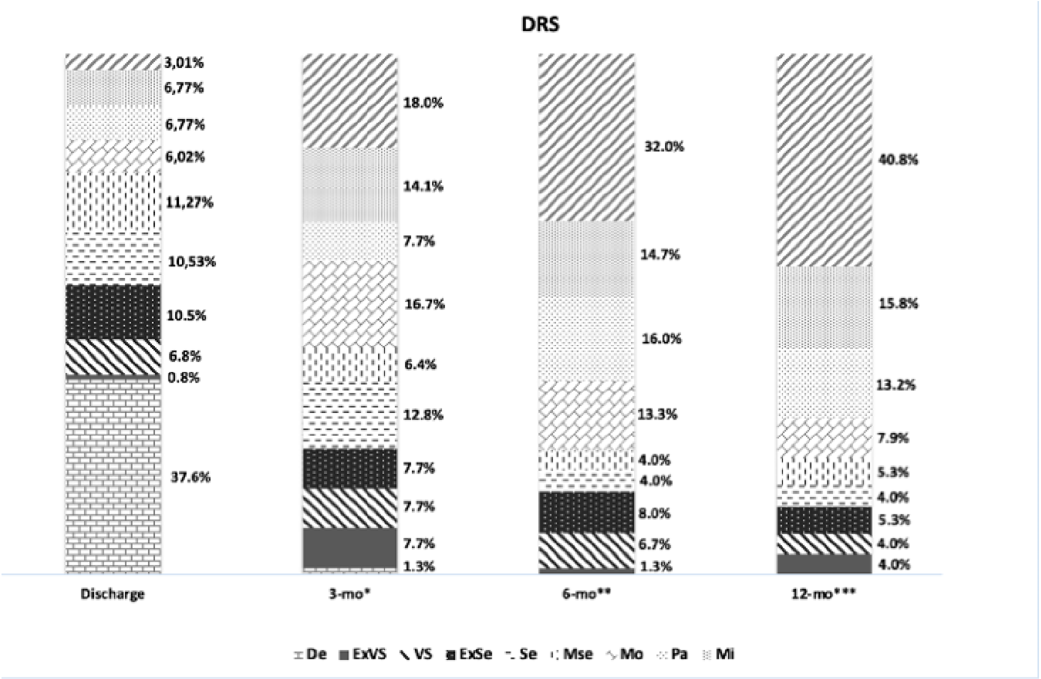

Figure S1. – DRS stratification at discharge, 3 months, 6 months and 12 months after TBI. De: Death; ExSe: Extremely Severe; ExVS: Extreme Vegetative State; GR-: Lower Good Recovery; GR+: Upper Good Recovery; MD-: Lower Moderate Disability; MD+: Upper Moderate Disability; Mi: Mild; Mo: Moderate; Mse: Moderately Severe; Pa: Partial; SD-: Lower Severe Disability; SD+: Upper Severe Disability; Se: Severe; VS: Vegetative State.
